# Everyday life following hematopoietic stem cell transplantation: decline in physical symptoms within the first month and change-related predictors

**DOI:** 10.1007/s11136-017-1705-3

**Published:** 2017-09-12

**Authors:** Aleksandra Kroemeke, Małgorzata Sobczyk-Kruszelnicka, Zuzanna Kwissa-Gajewska

**Affiliations:** 1 Department of Psychology, SWPS University of Social Sciences and Humanities, Chodakowska Street 19/31, 03-815 Warsaw, Poland; 2 Maria Sklodowska-Curie—Oncology Center, Gliwice Branch, Gliwice, Poland; 3 Department of Psychology, SWPS University of Social Sciences and Humanities, Warsaw, Poland

**Keywords:** Physical symptoms, Hematopoietic stem cell transplantation, Intensive longitudinal study, Multilevel modeling

## Abstract

**Purpose:**

Lower quality of life, especially in the physical domain (Physical-QOL), is common in patients after hematopoietic stem cell transplantation (HSCT). However, few studies explore changes in the Physical-QOL, i.e., physical symptoms, in everyday life of patients following HSCT. The present study addresses this gap by examining patient daily physical symptoms and their predictors in terms of demographic and clinical characteristics.

**Methods:**

Physical symptoms were reported by 188 patients (56.9% men; aged 47.6 ± 13.4 years) for 28 consecutive days after post-HSCT hospital discharge. Multilevel modeling was used to investigate fixed and random effects for physical symptom changes over time.

**Results:**

The results indicated that the initial level of physical symptoms (immediately after hospital discharge) systematically decreased over 28 days. Treatment toxicity (WHO scale; *β* = 0.09, *p* < .01) and baseline depressive symptoms (CES-D scale; *β* = 0.06, *p* < .01) were associated with the initial level of physical symptoms. Patients with more depressive symptoms before HSCT and with more adverse treatment effects presented with more physical symptoms immediately after hospital discharge. The type of transplant, diagnosis, and conditioning regimen differentiated the course of physical symptoms. Patients with leukemias and other myeloid neoplasms (*β* = 0.05, *p* < .01), after allogeneic HSCT (*β* = −0.06, *p* < .01), and with non-myeloablative conditioning (*β* = −0.09, *p* < .01) showed a significant lower decrease in symptoms over time. Patients with multiple myeloma presented with the most rapid improvement (*β* = −.03, *p* < .05).

**Conclusions:**

The findings suggest a heterogeneous and rather positive response to HSCT. Treatment-related conditions occurred to be a significant predictor of the intensity of change in physical functioning after HSCT.

## Introduction

Although high dose-therapy (chemotherapy and radiotherapy) and hematopoietic stem cell transplantation (HSCT) lead to an improved long-term survival of patients treated by these methods [1, 2], such treatments have an impact on the well-being of patients and the overall short- [3–6] and long-term quality of life [7–9]. The main treatment-related side effects of HSCT include a wide range of physical symptoms, such as loss of appetite, skin, eye and mouth problems, trouble sleeping, or fatigue [10, 11].

Adverse physical symptoms are common indicators of the health-related quality of life (HRQOL) physical domain [12]. Earlier studies suggest that poorer physical condition is associated with diminished well-being of patients [13], a lower psychological domain of HRQOL [14], higher distress, anxiety and depression [13, 15–18], and poorer health prognosis and survival rates [19–21]. Physical functioning of patients after HSCT is frequently the focus of studies. Still little is known about the *dynamics* of changes in physical symptoms or physical HRQOL over time after HSCT and the determinants of these changes. The present study addresses this gap.

The findings of previous studies indicate patient physical condition improves with time when considering the hospitalization period [4], first 14 days [5], 24 days [3, 17], and 100 days after HSCT [15]. However, studies over long periods of time indicate physical symptoms remain stable over a 3-year [22] or 9-year follow-up period [23]. Several factors are associated with better patient physical HRQOL following HSCT including younger age [8, 17, 18, 22], male gender [5, 8, 24, 25], employment [8], lack of comorbidities [18, 26], autologous (patient’s own stem cells) HSCT [18, 22], less-intensive previous therapy [18], lack of chronic graft-versus-host disease (GvHD; a medical complication following HSCT) [8, 9, 22], and lack of depression [27]. Similar predictors were identified in a recent meta-analysis indicating a strong effect of chronic GvHD, and weak and inconsistent findings of the remaining factors [28]. In one study, younger age and not receiving systemic immunosuppression was related to a decline in physical symptoms over 3 to 7+ years post-transplant among recipients of allogeneic (i.e., donor stem cells) HSCT [29]. Despite a number of important advantages of these studies, their weakness is often a long time period from HSCT and the examination of predictors of physical HRQOL instead of predictors of *changes* in symptoms or the growth curve of physical symptoms over time. In fact, improvements in physical HRQOL and regression of treatment-related side effects occur shortly after HSCT [4, 5, 17]. Moreover, cross-sectional studies allow only a determination of the variability between participants, but not the intra-individual variability (within-person). Longitudinal studies, on the other hand, rarely involve multiple occasions and use advanced statistical methods that allow characterizing within-person processes. Hence, the following questions remain unanswered: what the trajectory is and what is responsible for the dynamics of physical symptoms over time after HSCT.

Therefore, the aim of this study was to (1) identify the growth curve of physical symptoms in everyday life of patients during the first month (28 days) after post-HSCT hospital discharge, and (2) to determine the predictors and moderators of changes in physical symptoms in terms of demographic and clinical characteristics using a sophisticated modeling technique. To our knowledge, this study is the first to examine patterns of change in post-HSCT physical symptoms in everyday life of patients immediately after hospital discharge. The first month after hospital discharge could be a challenging period—the patient is not given all-day care that was guaranteed in hospital settings and complaints that were present during hospitalization may still be present. However, systematic changes in the physical condition of patients can be predicted. Thus, we expected a decrease in physical symptom level over the study period. The second aim was to evaluate whether demographics (age, gender, education, employment, marital, and economic status) and pre- and peri-HSCT clinical variables (primary diagnosis, time since diagnosis, medical comorbidities, type of transplant, conditioning regimen [preparatory treatment to HSCT of various intensities, from lower- to higher-intensity], treatment toxicity, and baseline depression) help to determine time changes of physical symptoms in patients. Based on previous studies, we hypothesized that the above factors could be related to the initial level of physical symptoms and the symptom growth curve.

## Methods

The inclusion criteria for the study were: (1) the first autologous or allogeneic HSCT, (2) age ≥18 years, (3) no history of other major disabling medical or psychiatric condition, and (4) written informed consent. The recruitment occurred in a single center after elective hospital admission for HSCT. The research procedure consisted of two stages: (1) baseline measurement (before conditioning regimen) in which demographic and medical characteristics, as well as patient-reported depression were measured; and (2) daily evening self-reported physical symptoms for 28 days starting from the first day of hospital discharge. Following recommendations for dyadic research [30], the format of the diary was adjusted to the individual preferences of the participants, namely: a traditional pen-and-paper form, an electronic version sent to a provided email account, or a telephone interview. This minimalizes the percentage of persons refusing to participate in the study and will not impact the results of a statistical analysis [31]. In this study, only paper (85.6%) and email modes (14.4%) were chosen by the participants. In the daily assessment, participants received a short text message (SMS) every evening to remind them to fill in their diary. Participation was voluntary. The study protocol was approved by the Ethics Committee of the SWPS University of Social Sciences and Humanities.

### Measures

#### Daily somatic symptoms

Physical symptoms based on EORTC QOL-C30 symptom scales [32], Larsen and Kasimatis [33] day-to-day physical symptoms scale, and additional symptoms related to HSCT (e.g., altered taste, mouth or eye complaints) were assessed for 28 consecutive days after hospital discharge to obtain a representative account of daily health status of patients after HSCT. The final scale consisted of a checklist containing 21 symptoms self-assessed in the evenings. The participants checked the symptoms they experienced during each day, responding to the instruction: “Today I have experienced the following symptoms (check all that apply).” The list of symptoms was as follows: dyspnea, tightness in chest, dizziness, nausea, vomiting, diarrhea, constipation, skin rush, numbness/tingling, pain (headache, backache, muscle soreness, other), dry/sore mouth and burning sensation in the mouth, altered/loss of sense of taste, burning/dry eyes, fatigue, trouble concentrating, insomnia, appetite loss, cough/runny nose. The participants had an option to add symptoms that were not included in the list. The narrative responses were compared against the symptoms on the scale. Symptoms that differed from the scaled items were added as “other” (i.e., the 22nd symptom). The daily physical symptoms score was calculated as the sum of experienced symptoms (total daily score: 0–22).

#### Sociodemographic and clinical characteristics

Demographic data included age, gender, education, marital status, subjective economic status, and employment. These data were collected by self-report before HSCT. Clinical data included diagnosis, time since diagnosis, comorbidities (number of comorbidities co-occurring with primary diagnosis), type of transplant, and conditioning. Clinical data were abstracted from medical records. Treatment toxicity was assessed by a physician using the World Health Organization (WHO) standard toxicity scale [34] at the end of hospitalization. The scale consists of 20 items (e.g., hemoglobin, leucocytes, creatinine, bilirubin, skin reaction, infection, cardiac function) related to the functioning of various organs and is assessed on a five-point scale from 0 (slight disturbance) to 4 (very high disturbance). Generally, the higher the result, the greater the toxicity of treatment (total score: 0–80). The internal consistency of the scale was 0.70. Self-reported depressive symptoms were assessed before HSCT with the 20-item Center for Epidemiological Studies Depression Scale (CES-D) [35] on a four-point scale from 0 (rarely or never) to 3 (often). The higher the result, the greater the number of depressive symptoms (total score: 0–60). The internal consistency of the scale was 0.87.

### Statistical analysis

We used an intensive longitudinal study procedure to test the study hypotheses. The essence of this method is to carry out more frequent (daily) measurements compared to traditional longitudinal studies. This new analysis allows a determination of the within-person change variability [31]. This procedure also minimizes the retrospective character of the data obtained during the study, which is typical of traditional research design [31, 36].

To identify the time course of somatic symptoms in post-HSCT patients, multilevel modeling (MLM) was conducted using IBM SPSS statistical package ver. 24. MLM provides the best parameter estimates while accommodating the hierarchical structure of the data (daily assessment nested within individuals) [31, 37]. An a priori power analysis using G*Power [38] with the correction described by Kish [37] was conducted to determine the minimum sample size required to detect small effect (*f* = 0.15) with *α* = 0.05 and power = .80. The minimum acceptable sample size was determined to be *N* = 144 participants.

To test moderating effects of demographic and clinical variables on within-patient variation in physical symptoms across 28 diary days (level 1), between-person predictors (level 2) were added to the model. All predictors were grand-mean centered; dummy codes were created for categorical variables [31, 37]. To avoid multicollinearity, potentially correlated predictors (type of transplant, diagnosis, conditioning) were tested separately. First, the model with all demographics and clinical variables was estimated except the type of transplant, diagnosis, and conditioning. A better fitting model was presented (model 1). Next, a separate model for the type of transplant (model 2), diagnosis (model 3 for leukemias and other myeloid neoplasms, model 4 for multiple myeloma, model 5 for lymphomas), and conditioning (model 6) were calculated with model 1 variables as covariates. Due to the percentage distribution of the type of transplant and conditioning, a comparison was made between patients with autoHSCT and the remaining types of alloHSCT in total and between myeloablative (MA; high-intensity) conditioning with non-myeloablative (NMA; low-intensity) and reduced intensity conditioning (RIC; intermediate-intensity) in total.

In all models, the restricted maximum likelihood (REML) was used as the estimator. Goodness of fit for the models was based on −2 Restricted log-likelihood ratio (−2LL), the Akaike Information Criterion (AIC), and the Bayesian Information Criterion (BIC). The first-order autoregressive [AR(1)] covariance structure was used for the models, given the common proximal autocorrelation in the daily data [39].

## Results

### Sample characteristic

A total of 437 patients met the study criteria between November 2014 and November 2016. Of the 437 eligible patients, 238 gave their written informed consent and filled in the baseline measurement questionnaire (1, stage). The final sample included 188 participants who participated in the daily study for at least 7 days (2, stage). Most participants were in a stable relationship, had at least a secondary education, were professionally inactive, and assessed their economic status as average and underwent autologous HSCT (autoHSCT) and myeloablative conditioning (high-intensity conditioning; see Table 1).

**Table 1:** Sample characteristics

Demographic characteristics	Patients (*N* = 188)
	*n* (%)
Male	107 (56.9%)
White race	188 (100%)
Employment: yes	72 (38.5%)
Marital status	
Single	19 (10.1%)
Married/partnership	158 (84%)
Divorced/widowed	11 (5.9%)
Subjective economic status	
Above average	26 (13.8%)
Average	150 (79.8%)
Below average	12 (6.4%)

	*M* (*SD*), range
Age (years)	47.62 (13.37), 19–68
Education (years)	14.39 (3.30), 6–28

Clinical characteristics	Pre- and peri-transplant period
	*n* (%)
Primary diagnosis	
Leukemias and other myeloid neoplasms	36 (19.2%)
Acute leukemia (ALL, AML)	30 (16%)
Chronic leukemia (CML)	3 (1.6%)
Myelodysplastic syndrome (MDS)	1 (0.5%)
Myeloproliferative disorders	2 (1.1%)
Lymphomas	84 (44.7%)
Hodkin (HL)	21 (11.2%)
Non-Hodkin (NHL)	63 (33.5%)
Multiple myeloma (MM)	58 (30.8%)
Other cancer types (solid tumor, other)	10 (5.3%)
Medical comorbidities	
None	82 (43.6%)
1	59 (31.4%)
2	26 (13.8%)
3 or more	21 (11.2%)
Type of transplant	
Autologous (autoHSCT)	139 (74%)
Allogeneic (alloHSCT)	49 (26%)
Matched sibling donor	35 (18.6%)
Matched unrelated donor	10 (5.3%)
Haploidentical	4 (2.1%)
Conditioning	
Myeloablative (MA)	177 (94.2%)
Non-myeloablative (NMA)	7 (3.7%)
Reduced intensity (RIC)	4 (2.1%)
Acute GvHD (only alloHSCT)	20 (40.8%)

	*M* (*SD*), range
Time since diagnosis (months)	20.96 (23.53), 3–180
Medical comorbidities	0.99 (1.18), 0–6
Days from HSCT to discharge	18.44 (9.02), 10–91
AutoHSCT recipients	14.89 (7.19), 10–91
AlloHSCT recipients	27.98 (6.04), 17–45
Treatment toxicity (WHO scale)	18.09 (4.87), 0–37
Depressive symptoms before HSCT (CES-D scale)	16.51 (8.51), 3–42

MA—conditioning regimen that produces irreversible pancytopenia and requires stem cell support; NMA—conditioning that produces minimal pancytopenia and does not require stem cell support; RIC—conditioning regimen that does not fulfill MA or NMA definition [39]

*ALL* acute lymphoblastic leukemia, *AML* acute myeloid leukemia, *CML* chronic myelogenous leukemia, *GvHD* graft-versus-host disease

Of the remaining 50 participants, 6 were disqualified from HSCT, 16 died at the time of isolation, 28 resigned from the daily assessment. Sample attrition analyses (using binomial logistic regression) indicated that the daily study completers and non-completers did not differ in terms of sociodemographic (age, gender, education, marital status, economic status, and employment), health-related variables (diagnosis, time since diagnosis, comorbidities, conditioning), or depressive symptoms at baseline, except treatment toxicity (*B* = −0.06, *SE* = 0.02, *p* = .009, OR = .94), and type of transplant (*B* = −1.34, *SE* = 0.33, *p* < .001, OR = .26). Higher treatment toxicity according to the WHO Toxicity Scale and allogeneic HSCT (alloHSCT) were associated with an increased likelihood of belonging to the non-completers group.

### Missing data analysis

The number of missing observations amounted to 9% (across all days and participants; from 2.7% on day 1–13.3% on day 28), with 61% of fully completed diaries. There were no significant associations between missing data and demographic characteristics, clinical variables, daily physical symptoms, and belonging to the paper or an email group. The data were missing at random. MLM leads to unbiased estimates in that case [37]. The final analysis dataset consisted of 4780 daily reports from 188 patients.

### Growth curve of daily somatic symptoms

Descriptive statistics of daily physical symptoms are presented in Table 2. The MLM analysis indicated that the initial level of physical symptoms (intercept; immediately after hospital discharge) was 4.3 unit on a 0–22 scale and showed a 0.06 unit decrease over time (slope; 28 days; Cohen’s *d* effect size = 1.28); −2LL = 15365.59, AIC = 155375.59, BIC = 15407.95. Besides, there was evidence of between-person (i.e., between subject) variability in both the intercept (*B* = 6.40, *SE* = 0.02, *p* < .001) and slope (*B* = 0.01, *SE* = 0.00, *p* < .001) of physical symptoms. The intraclass correlation coefficient (ICC) was 0.72, also indicating between-person differences in the physical symptoms.

**Table 2:** Descriptive statistics of self-reported daily physical symptoms (*N* = 188 participants)

Day	*M*	*SD*	Min.	Max.	Day	*M*	*SD*	Min.	Max.
Day 1	4.81	3.11	0	15	Day 15	3.23	2.64	0	13
Day 2	4.34	2.94	0	15	Day 16	3.17	2.53	0	13
Day 3	4.29	2.88	0	16	Day 17	3.13	2.61	0	14
Day 4	4.12	2.69	0	15	Day 18	3.35	2.77	0	14
Day 5	3.99	2.85	0	16	Day 19	3.26	2.89	0	14
Day 6	3.98	2.72	0	13	Day 20	3.17	2.76	0	14
Day 7	3.74	2.69	0	13	Day 21	3.27	2.91	0	13
Day 8	3.85	2.72	0	12	Day 22	3.13	2.76	0	12
Day 9	3.67	2.60	0	12	Day 23	3.11	2.65	0	11
Day 10	3.53	2.49	0	13	Day 24	3.23	2.71	0	12
Day 11	3.29	2.59	0	13	Day 25	3.02	2.55	0	12
Day 12	3.35	2.75	0	13	Day 26	2.96	2.52	0	12
Day 13	3.30	2.59	0	13	Day 27	2.90	2.42	0	12
Day 14	3.26	2.56	0	13	Day 28	2.75	2.49	0	13

Daily physical symptoms were assessed using a self-assessed checklist containing 21 symptoms and the “other” option (22nd symptom). The scores were calculated as the sum of experienced symptoms (total daily score: 0–22)

### Predictors of time course of daily somatic symptoms

Preliminary results of MLM indicated that only treatment toxicity (*B* = 0.11, *SE* = 0.04, *p* = .008) and pre-HSCT depressive symptoms (*B* = 0.05, *SE* = 0.02, *p* = .022) were significantly associated with the intercept of physical symptoms, but did not differentiate the slope of physical symptoms (*B* = −0.01, *SE* = 0.00, *p* = .832; *B* = 0.00, *SE* = 0.00, *p* = .819 for WHO and CES-D scale, respectively). The remaining variables and their interactions with time were not statistically significant. Final model 1 included treatment toxicity and depression, controlling for the age, gender, and comorbidities. Patients with more depressive symptoms before HSCT and with more adverse treatment effects had more physical symptoms immediately after hospital discharge. The results from MLM (final models) examining associations between level two variables as predictors of time course of physical symptoms are given in Table 3.

**Table 3:** Estimates of multilevel models: daily physical symptoms as a function of demographic and clinical characteristics—final models (*N* = 4780 observations)

	Model 1 basic model	Model 2 autologous HSCT (autoHSCT)	Model 3 leukemias and other myeloid neoplasms	Model 4 multiple myeloma (MM)	Model 5 lymphomas	Model 6 myeloablative (MA) conditioning
Fixed effects, estimate (*SE*)						
Intercept for Day 1	4.37 (0.24)***	4.24 (0.42)***	4.63 (0.79)***	4.36 (0.89)***	4.46 (0.80)***	3.68 (0.1.21)***
Days, centered at Day 1	−0.06 (0.01)***	−0.02 (0.01)	−0.07 (0.01)***	−0.05 (0.01)***	−0.06 (0.01)***	0.03 (0.03)
Age	0.02 (0.01)	0.02 (0.01)	0.02 (0.01)	0.02 (0.01)	0.02 (0.01)	0.02 (0.01)
Gender (1 = female, 0 = male)	−0.16 (0.34)	−0.21 (0.35)	−0.18 (0.35)	−0.18 (0.35)	−0.19 (0.35)	−0.17(0.35)
Comorbidity	0.20 (0.16)	0.21 (0.16)	0.20 (0.16)	0.20 (0.16)	0.20 (0.16)	0.21 (0.16)
Treatment toxicity (WHO)	0.09 (0.03)**	0.08 (0.03)*	0.09 (0.03)*	0.09 (0.03)*	0.09 (0.03)*	0.09 (0.03)**
Depressive symptoms (CES-D)	0.06 (0.02)**	0.06 (0.02)**	0.06 (0.02)**	0.06 (0.02)**	0.06 (0.02)**	0.06 (0.02)**
AutoHSCT		0.21 (0.44)				
AutoHSCT × Days		−0.06 (0.02)***				
Leukemias and other myeloid neoplasms			−0.55 (0.88)	0.10 (0.85)	0.09 (0.85)	
Leukemias and other myeloid neoplasms × Days			0.05 (0.02)**			
MM			−0.19 (0.85)	0.25 (0.87)	−0.17 (0.85)	
MM × Days				−0.03 (0.01)*		
Lymphomas			−0.18 (0.81)	−0.17 (0.81)	−0.09 (0.83)	
Lymphomas × Days					−0.01 (0.01)	
MA conditioning						0.74 (1.21)
MA conditioning × Days						−0.09 (0.03)**
NMA + RIC						−0.13 (0.1.46)
Random effects, estimate (*SE*)						
Residual	1.60*** (0.05)***	1.60 (0.05)***	1.60*** (0.05)***	1.60*** (0.05)***	1.60*** (0.05)***	1.60*** (0.05)***
Autocorrelation (AR1)	0.51*** (0.02)***	0.51 (0.02)***	0.51*** (0.02)***	0.51*** (0.02)***	0.51*** (0.02)***	0.51*** (0.02)***
−2LL	15313.80	15303.99	15309.97	15313.65	15318.36	15305.32
AIC/BIC	15323.80/15356.14	15313.99/15346.34	15319.97/15352.33	15323.65/15355.99	15328.36/15360.70	15315.32/15347.66

Unstandardized estimates and standard errors (*SE*)

The interpretation of the MLM based on the example of model 2: (1) the intercept is the level of physical symptoms on Day 1 after hospital discharge for the alloHSCT group, (2) the Days estimate is the change in physical symptoms in the alloHSCT group over 28 days of the study, (3) the autoHSCT estimate is the difference in somatic symptoms (auto- minus alloHSCT) on Day 1, (4) the autoHSCT-by-day interaction is a difference in somatic symptoms change between allo- and autoHSCT groups

*NMA* non-myeloablative conditioning, *RIC* reduced intensity conditioning, *−2LL* −2 restricted log-likelihood ratio, *AIC* the Akaike information criterion, *BIC* the Bayesian information criterion

*p < .01, **p < .01, ***p < .001

**Fig. 1:**
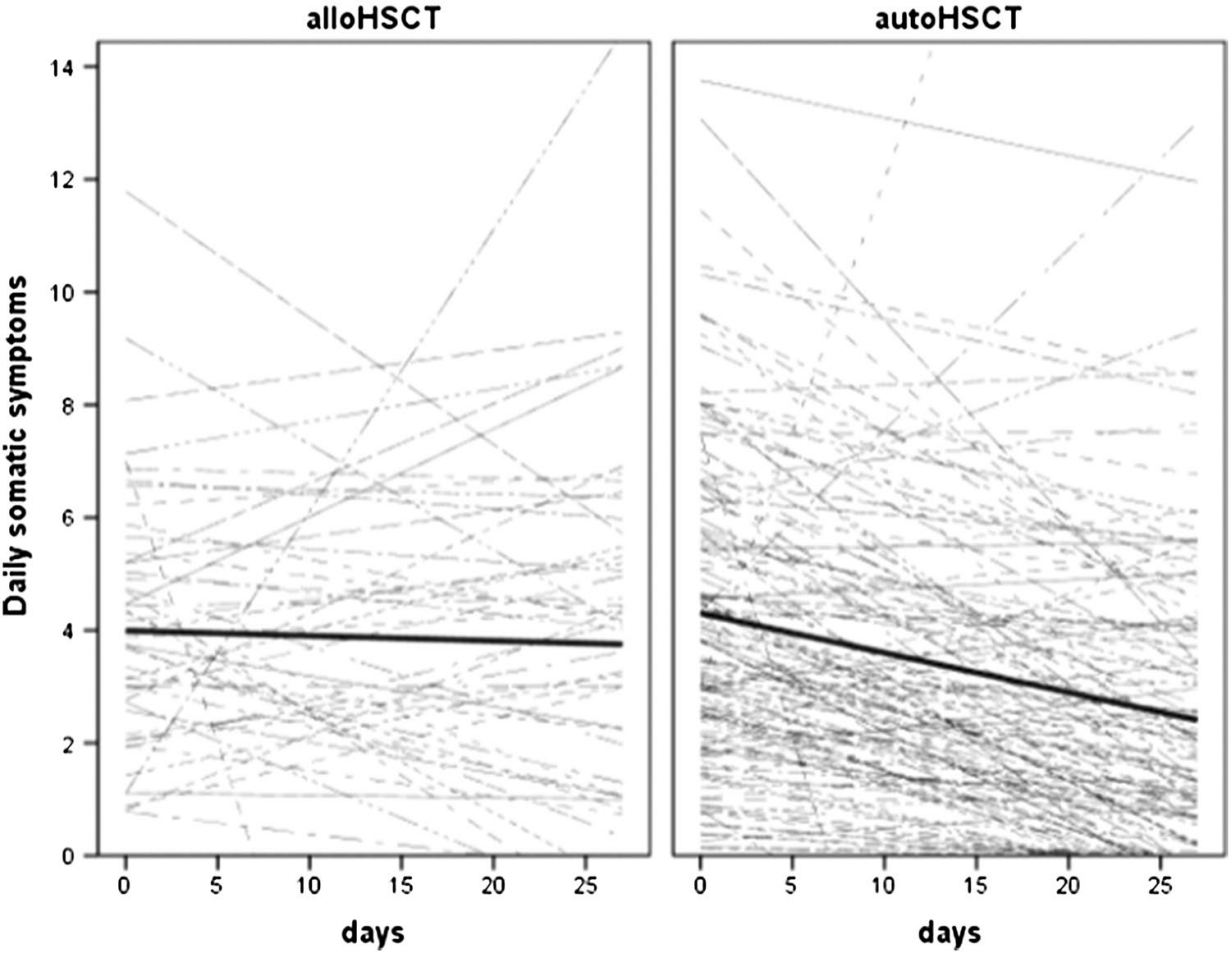
Spaghetti plot of average (thick) and patient-specific (thin) time courses of somatic symptoms for alloHSCT (left) and autoHSCT (right) groups

The model 2 parameters (moderator effect of type of transplant: 1 = autoHSCT, 0 = alloHSCT, controlling for the age, gender, comorbidities, treatment toxicity, and pre-HSCT depression) showed that there were no group differences in the initial level of physical symptoms. The autoHSCT-by-day interaction indicated group differences in time course of physical symptoms. AlloHSCT group did not show physical symptom change over 28 days of the study (non-significant 0.02 unit decrease), whereas the autoHSCT group showed their 0.08 unit decrease over time (see Fig. 1).

**Fig. 2:**
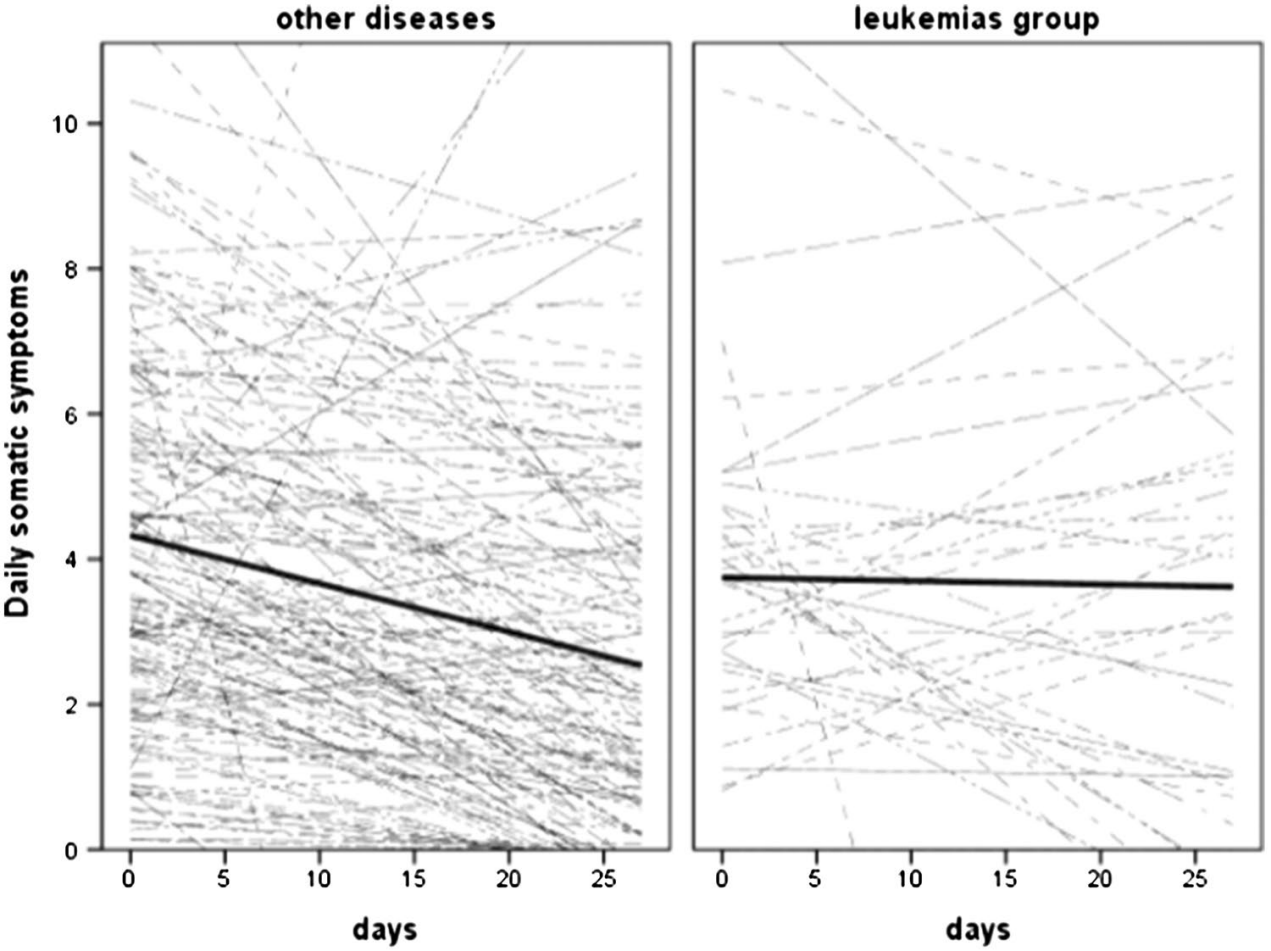
Spaghetti plot of average (thick) and patient-specific (thin) time courses of somatic symptoms for other diseases (left) and leukemias and other myeloid neoplasms (right)

The result of model 3 examining the moderator effect of leukemias and other myeloid neoplasms (1 = leukemias group, 0 = other diseases), controlling for the age, gender, comorbidities, treatment toxicity, and pre-HSCT depression, indicated no group difference in the intercept of physical symptoms. However, there was a significant leukemias-by-day interaction i.e., patients with leukemias and other myeloid neoplasms showed only a 0.02 unit decrease in physical symptoms in time compared to other patients with a significant 0.07 unit decrease (see Fig. 2).

**Fig. 3:**
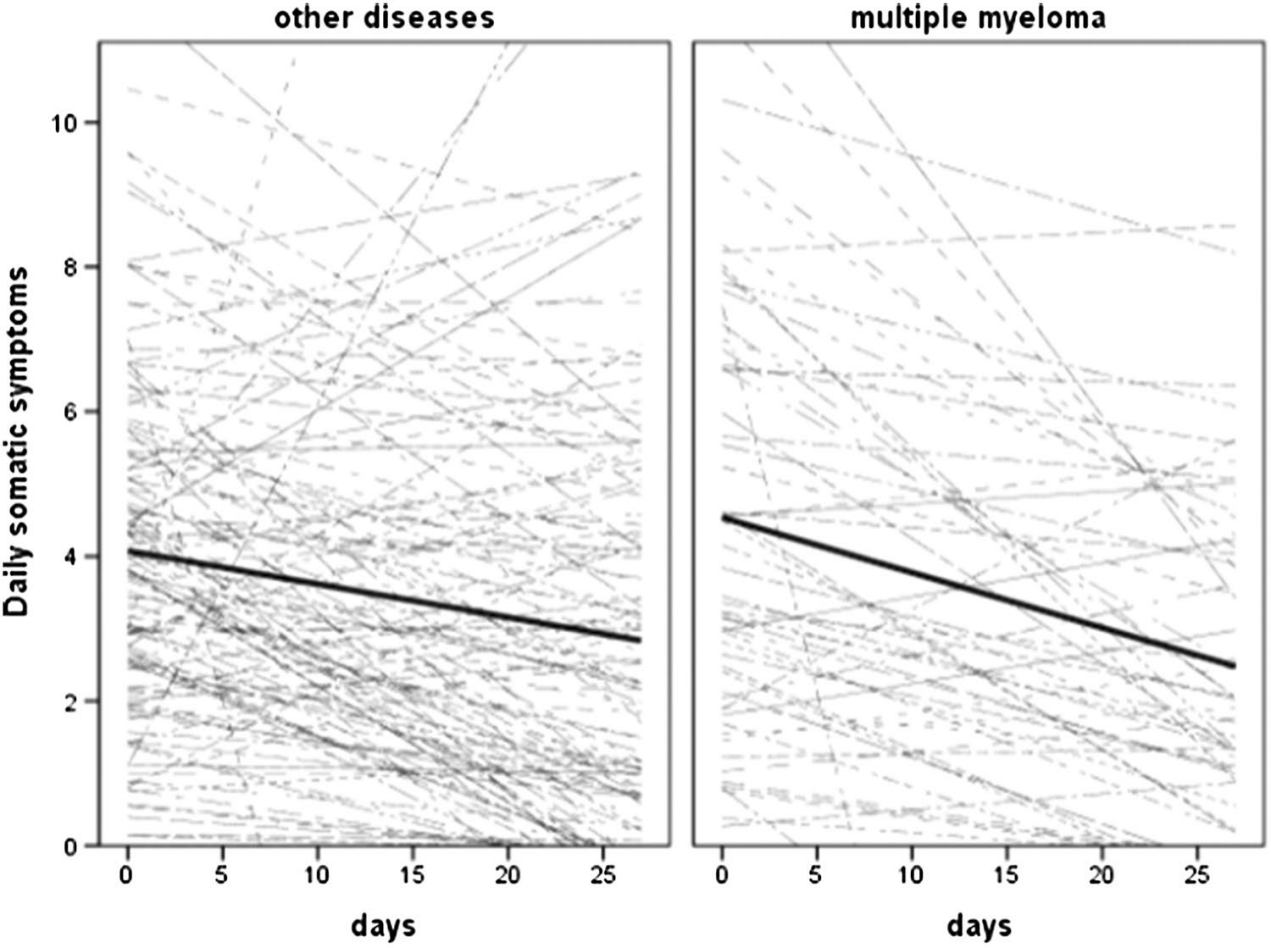
Spaghetti plot of average (thick) and patient-specific (thin) time courses of somatic symptoms for other diseases (left) and multiple myeloma (right)

The result of model 4 examining the moderator effect of multiple myeloma (1 = MM, 0 = other diseases), controlling for the age, gender, comorbidities, treatment toxicity, and pre-HSCT depression, revealed significant group differences in the growth curve of physical symptoms (see Fig. 3). The decrease in physical symptoms over 28 days in the MM group was stronger (0.08) than in non-MM group (0.05 unit decrease).

**Fig. 4:**
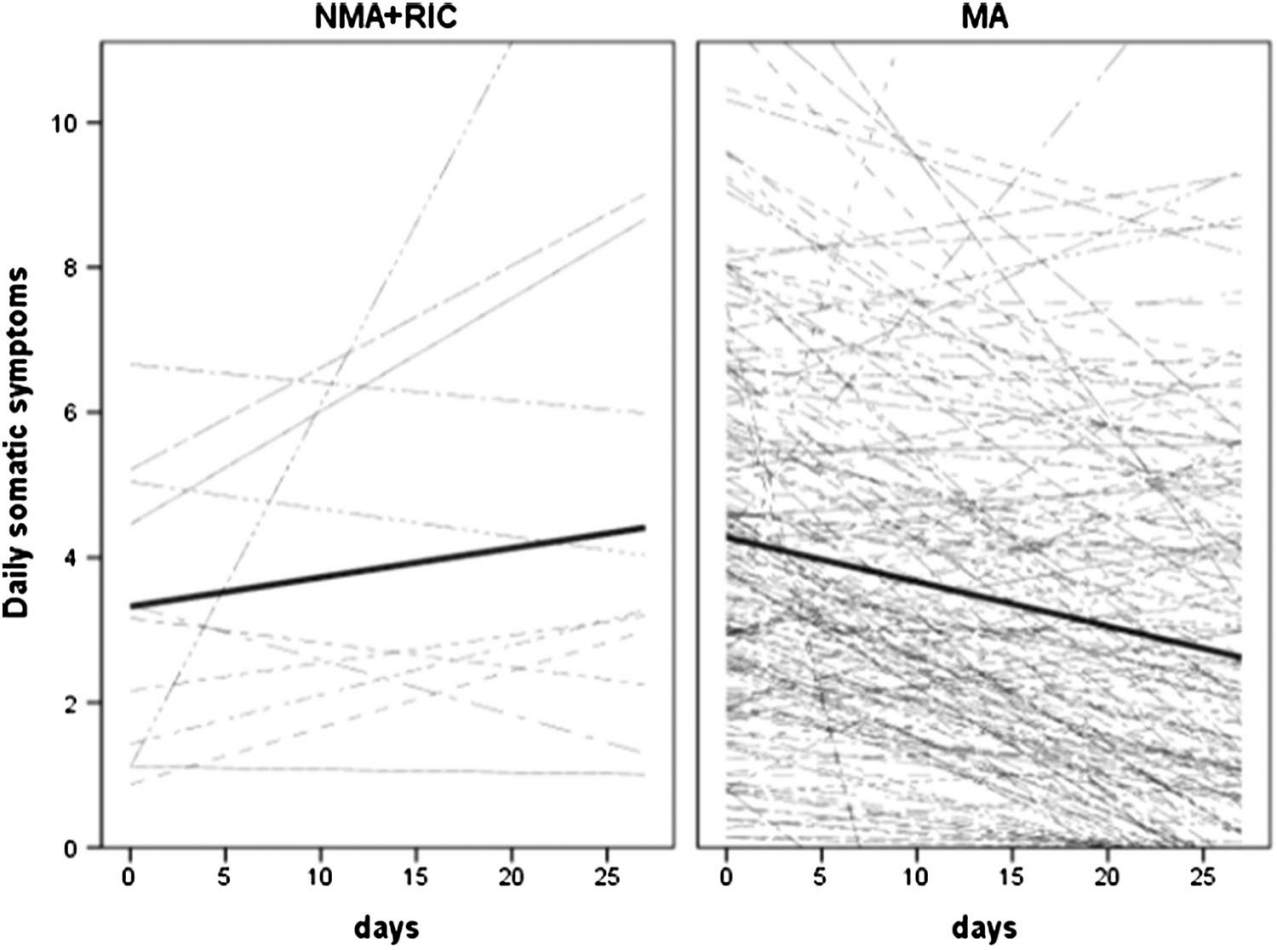
Spaghetti plot of average (thick) and patient-specific (thin) time courses of somatic symptoms for non-myeloablative (NMA) + reduced intensity (RIC) (left) and myeloablative (MA) conditioning (right) groups

Finally, the result of model 6 examining the moderator effect of conditioning (1 = MA, 0 = NMA + RIC), controlling for the age, gender, comorbidities, treatment toxicity, and pre-HSCT depression, indicating a significant difference in the growth curve of physical symptoms between MA and non-MA group (see Fig. 4). MA patients reported a 0.06 unit decrease in somatic symptoms over time, whereas in non-MA patients, symptoms were stable over time (non-significant 0.03 unit increase).

No moderator effect of lymphomas was found (see model 5). Besides, as it was expected, there was evidence of autocorrelation in the level 1 residuals in all models.

## Discussion

Due to the lack of longitudinal research on the time course of physical HRQOL in post-HSCT patients, the aim of this study was to examine the time-based trajectory of their physical symptoms over 28 days and trajectory determinants. The findings revealed a significant systematic decrease in physical symptoms in time, moderated by the type of disease, transplant, and preparatory treatment (conditioning).

Immediately after hospital discharge patients reported on average 4 out of 22 possible symptoms and their number decreased in time. This indicates a relatively good physical adaptation to HSCT as evidenced by few treatment-related side effects and symptom resolution within the first month after hospital discharge. Such results are consistent with the current longitudinal studies conducted in a similar time period and in the traditional mode [3–5, 17]. The decline in symptoms in the current study was clinically significant as indicated by large effect size index. Significant between-person differences were also noted for the initial level and the time trend of physical symptoms. The range of the results was 0–15 symptoms and was present for all 28 days.

Since the group was heterogeneous in terms of physical symptoms, we tested for the moderator responsible for this variability. Significant independent predictors of the initial level of physical symptoms were treatment toxicity and baseline depression, after adjusting for the effects of age, gender, and comorbidities.

The more toxic the treatment as measured by the WHO scale, the more symptoms were reported by patients on Day 1 of the study. These important findings were not previously reported in the literature. Moreover, a higher initial level of physical symptoms was also related to higher pre-HSCT depressive symptoms. Positive relationships between these variables were observed in previous studies [13, 27, 28]. One explanation for this effect is that depression is associated with increased adverse symptom burden [40] via pathophysiologic processes (e.g., increased cytokine level or other inflammatory factors) [41]. Likewise, depression or depressive symptoms are related to the increased perception and the focus on physical symptoms [40]. Patients with elevated depressive symptoms may be more “sensitive” to certain symptoms such as pain or altered taste. Of note, our analyses were related to baseline depressive symptoms. However, previous studies reported that pre-HSCT depressive symptoms predicted their level in post-HSCT period [5, 25]. Therefore, it is probable that patients with a larger number of depression symptoms prior to HSCT experienced the increased level of symptoms also in the further period. Finally, reported physical symptoms may be a part of depressive mood. Depressive patients may report more symptoms, such as fatigue, lack of concentration, or sleep-related problems, which may be connected to the patient mood and not to the adverse effects of treatment. These findings have significant clinical implications and highlight the two important risk factors for a higher level of physical symptoms (or lower physical HRQOL) at discharge: treatment toxicity and baseline depression. They also identify a significance for patient screening for mood disorders and the provision of psychological care to patients with lowered mood as early as prior to HSCT.

Interestingly, neither treatment toxicity nor baseline depression determined the further course of symptoms in time. Significant moderators of change were type of disease, type of transplant, and conditioning regimen, after controlling for the age, gender, comorbidities, treatment toxicity, and baseline depression. Patients with autoHSCT, MA conditioning, and with MM were characterized by a significant higher decrease in symptoms over time. In turn, patients with leukemias and other myeloid neoplasms demonstrated a significantly lower decrease in symptoms over time as compared to the remaining patients. A better physical HRQOL among autoHSCT recipients as compared to alloHSCT patients was reported in previous studies [5]. A novel finding in our study is a more rapid resolution of adverse effects after autoHSCT, hence the improvement in physical HRQOL in this patient group.

Patients with leukemias and other myeloid neoplasms experienced lower decrease in treatment-related adverse symptoms. Previous studies also reported poorer functioning of patients with leukemias as compared to MM and lymphoma patients [28]. Poorer functioning may be due to immunosuppression, allogeneic transplant, and disease connected with high-intensity treatment before HSCT.

Patients with MM experienced the most rapid improvement in adverse physical symptoms. The similar result was reported in another study [10]. Faster improvement in the physical symptoms of patients with MM may be related to the autologous type of transplant or MM itself. These patients usually have several different (mainly pain-related) complaints. Some complaints may resolve in the post-HSCT period as a result of discontinuation of neurotoxic drug treatment.

Patients with lymphomas were similar to patients with leukemias, other myeloid neoplasms, and MM in terms of the level and time course of physical symptoms. Our findings highlight the difference in adverse symptom change among and between various disease groups and awareness about the diversity of recovery of patients which can prevent stereotyping and routine approaches to their physical problems.

Somewhat surprisingly, our results show conditioning regimen associated with higher toxicity (MA) [42] also resulted in a more rapid decrease in symptoms over time. Comparably, Andersson et al. [11] found a faster increase in the physical symptoms in alloHSCT recipients who had undergone RIC compared to MA conditioning; however, the timeframe of both studies does not allow the comparison between them. When interpreting the obtained results, attention should also be given to the interactions of the mentioned clinical characteristics. Among MA patients, the predominant patients were those after autoHSCT (73%), diagnosed with MM (31%) and lymphomas (44%). Of note, autoHSCT model was the best-fitting model (see Table 3 AIC and BIC values).

Our study has a number of limitations. First, significant disproportions in the number of members in the compared groups (MA vs. non-MA) could have introduced bias into our results. One of the remaining factors such as type of transplant may be the cause of the MA effect. The issue of MA-related long-term adverse symptoms requires further investigation. Second, there were a disproportionate number of patients who received autoHSCT in the study group. Hence, larger groups of patients with different conditioning regimen and type of transplant should be enrolled in future studies. Third, depressive symptoms were not controlled on a daily basis, which could have the interpretative significance. Fourth, depressive symptoms were based on self-report assessments rather than clinical diagnosis. Finally, we tested only the moderator effect of demographic and clinical factors on the initial level at discharge and the time course of physical symptoms.

Due to the heterogeneity of the group, it would also be reasonable to examine whether it is possible to identify in the study group the patient subgroups with the similar baseline level and trajectory of symptoms in time, in accordance with the person-centered approach [43]. Despite the limitations, the current study is the first to use an intensive longitudinal approach to examine changes in physical symptoms or physical HRQOL over the first month following HSCT. The findings highlight the heterogeneity of the growth curve of physical symptoms and the manner in which several clinical factors are associated with the change in symptoms, indicating the practical implications of these results.
